# Biosynthesis and Characterization of Silver Nanoparticles from the Extremophile Plant *Aeonium haworthii* and Their Antioxidant, Antimicrobial and Anti-Diabetic Capacities

**DOI:** 10.3390/nano13010100

**Published:** 2022-12-25

**Authors:** Badiaa Essghaier, Rihab Dridi, Filomena Mottola, Lucia Rocco, Mohamed Faouzi Zid, Hédia Hannachi

**Affiliations:** 1 Department of Biology, Faculty of Sciences, University of Tunis El-Manar II, Tunis 2092, Tunisia; 2 Laboratoire de Matériaux Cristallochimie et Thermodynamique Appliquée, Department of Chemistry, Faculty of Sciences of Tunis, University of Tunis El-Manar II, Tunis 2092, Tunisia; 3 Department of Environmental Biological and Pharmaceutical Sciences and Technologies (DiSTABiF), University of Campania L.Vanvitelli, 81100 Caserta, Italy; 4 Laboratory of Vegetable Productivity and Environmental Constraint LR18ES04, Department of Biology, Faculty of Sciences, University Tunis El-Manar II, Tunis 2092, Tunisia

**Keywords:** silver nanoparticles, *Aeonium haworthii*, antioxidant, antimicrobial, antidiabetic capacities

## Abstract

The present paper described the first green synthesis of silver nanoparticles (AgNPs) from the extremophile plant *Aeonium haworthii*. The characterization of the biosynthesized silver nanoparticles was carried out by using UV-Vis, FTIR and STM analysis. The antioxidant, antidiabetic and antimicrobial properties were also reported. The newly described AgNPs were spherical in shape and had a size of 35–55 nm. The lowest IC50 values measured by the DPPH assay indicate the superior antioxidant behavior of our AgNPs as opposed to ascorbic acid. The silver nanoparticles show high antidiabetic activity determined by the inhibitory effect of α amylase as compared to the standard Acarbose. Moreover, the AgNPs inhibit bacterial growth owing to a bactericidal effect with the MIC values varying from 0.017 to 1.7 µg/mL. The antifungal action was evaluated against *Candida albicans*, *Candida tropicalis*, *Candida glabrata*, *Candida sake* and non-dermatophytic onychomycosis fungi. A strong inhibitory effect on Candida factors’ virulence was observed as proteinase and phospholipase limitations. In addition, the microscopic observations show that the silver nanoparticles cause the eradication of blastospores and block filamentous morphogenesis. The combination of the antioxidant, antimicrobial and antidiabetic behaviors of the new biosynthesized silver nanoparticles highlights their promising use as natural phytomedicine agents.

## 1. Introduction

In nano-biotechnology, the biosynthesis of silver nanoparticles attracted great attention owing to their specific biophysical properties and enriched biocompatibility as well as their significant biological properties in biomedical, industrial, agricultural, food processing and environmental fields [1]. To avoid the drawbacks of physical and chemical nanoproducts synthesis methods, the extracts of medicinal plants have been widely used in the green synthesis of AgNPs as reducing/stabilizing agents. It is well known that the plant has promising medicinal significance due to the presence of diverse bioactive compounds, including flavonoids, phenolic acids, terpenoids, and alkaloids, useful for the biosynthesis of silver nanoparticles since phytochemicals in plants extracts help to reduce silver ions (Ag+) for synthesizing biomolecule encapsulated AgNPs.

Recently we have reported silver nanoparticles biosynthesis from Mediterranean species, including *Anagallis monelli* and *Scabiosa atropurpurea* subsp. *Maritima.* Both silver nanoparticles have shown specific antioxidant, antimicrobial and anticancer properties as compared to other published silver nanoparticles obtained from other species [2,3]. 

However, numerous plant species are known to exhibit a significant amount of antioxidant enzymes that could be exploited to improve the synthesis of silver nanoparticles. 

In the last decades, 12 non-native succulent taxa have been reported in the whole Mediterranean area or in North Africa and Tunisia. In fact, the cultivation of the ornamental plant species *Aeonium haworthii* caused its naturalization in other areas. *Aeonium haworthii* is one of the non-native succulent taxa reported in Tunisia and in Italy [4]. These taxa are belonging to the Crassulaceae family [5]. Recently, we discussed the use of the leaves and aerial parts of plants as a natural source of bioactive compounds used for the biosynthesis of silver nanoparticles (AgNPs) [6]. In this context, we have chosen an extremophile plant species to research new biogenic AgNPs. By adapting a simple one-step approach for AgNP synthesis using the aqueous extract from *Aeonium haworthii*, we characterize their structural and biological properties as potent nanomedicines. To the best of our knowledge, this is the first report of silver nanoparticles using an aqueous extract from *Aeonium haworthii*.

Silver nanoparticles possess significant biological properties such as antimicrobial, anti-inflammatory, antidiabetic, antioxidant, anticancer and antiviral effects [7]. Recently, several more biocompatible nanomaterials have been manufactured based on the incorporation of NPs, in order to accelerate their biomedical properties. For example, the concept of the release of metal ions has been explored for accelerating biomedical applications [8]. In this context, we find the work of Zhou et al., who describes a dental material composed of metal ion-incorporated NPs. This dental material shows an inhibiting effect against dental caries and biofilm as well as the improvement of the enamel hardness and decreases enamel demineralization [9]. Similar to the works of Kumar et al, who reported a design of PVP alcohol and chitosan-loaded AgNPs hydrogels, this composite shows superior antibacterial action, biocompatibility for wound dressing and healing of second-degree burns [10]. Moreover, Paterson et al. describe a new biocompatible implant material made of copper-containing glass NPs with both antibacterial and proangiogenic properties for chronic wounds by the release of Cu^2+^ ions [11].

Similar to the works of Singla and Coll, they mentioned the application of the nanocomposite AgNPs into cellulose in in vivo models; the skin of male Swiss albino mice exhibits antibacterial activity and accelerates wound healing [12].

Regarding the multiple richness of plants with polyphenols, these compounds can help prevent several diseases related to oxidative stress, such as cancer, and cardiovascular disease owing to their anti-inflammatory and antioxidant capacities. Based on the antioxidant capacity of plant extracts, they can be a good alternative to several resistant microbes [13,14]. Antioxidants have significant roles in tissue damage in various human diseases such as inflammation and cancer. Having antioxidant capacity associated with pharmaceutical properties such as anti-diabetic, and anti-inflammatory can be special to obtaining multifunctional drugs. The potential of nanotechnologies has been reported in biomedicine for the diagnosis and treatment of many human diseases [15]. Silver nanoparticles, owing to their physicochemical and nanostructure properties, antimicrobial capability, and low toxicity for mammalian cells, enhance their use in nanomedicine [16].

Referring to the encouraging applications of nanoparticles and nanocomposites as safe alternatives in various fields, numerous studies have targeted the toxicokinetic effects of silver nanoparticles, e.g., [17]. For example, the pretreatment of the kidney and liver in animal models (Sprague Dawley rats) with AgNPs at 2000mg/kg, indicates the low toxicity of the AgNPs by detecting the absence of abnormal reactions and minimal effects on treated organs [18].

Nanotechnology has been particularly important in the diagnosis and treatment of diabetes [19]. Non-insulin-dependent diabetes mellitus consists of 90% of diabetes. Alpha-glucosidase and pancreatic alpha-amylase play a crucial role in carbohydrate digestion and glycoprotein processing. For the oral treatment of non-insulin diabetes mellitus type II, enzyme inhibitors can be applied as regulators of carbohydrate absorption.

Synthetic antidiabetic drugs such as acarbose, voglibose, and metformin suppress the enzymes, but they have side effects such as diarrhea, bloating and distention [20]. In light of these data, researchers directed toward the discovery of new natural drugs to overcome the side effects. Considering these data, the extremophile plants possess significant phytoconstituents with relevant antioxidant capacities, related to their adaptation to extreme environmental conditions. 

Medicinal plants containing antioxidants and antidiabetic compounds have become increasingly popular for the management of diabetes because of their low cost and wide availability and lack of side effects [21]. Moreover, among the hypotheses proposed to explain hyperglycemia, is the disorder in the equilibrium between reactive oxygen species’ capacity and antioxidant defense capacity [22]. Based on these data, the application of antioxidant agents can be helpful for scavenging various reactive oxygen species and the prevention of diabetes mellitus [23]. In this context, in the present work, we describe the antioxidant and the hypoglycemic effect of the new biosynthesized silver nanoparticles by evaluating their in vitro inhibition of alpha-amylase as natural oral therapeutic drugs. In fact, the huge biomedical applications of the nanoparticles were correlated to their structural properties specifically the surface charge, size, morphology and high compatibility [24].

Regarding the impact of multiresistant bacteria and fungi human strains on the commonly used antimicrobial drugs on public health, increasing costs and hospitalization, these problems attract rising medicinal attention. Moreover, silver ions and silver nanoforms possess bactericidal and fungicidal effects against resistant strains by having different mechanisms of action against cell pathogens [25,26]. In regards to the pharmacology and toxicology fields, there is a critical need for non-toxic, more effective antimicrobial agents against bacterial infections, especially MDR clinical strains. In this context, the work of Roy et al. reported that skin exposed to AgNPs has no alteration or change in appearance, nor in the structure of treated skin; these findings indicate that the green, biosynthesized AgNPs provide a non-toxic rapid method to develop more effective and safe antimicrobial drugs for use as an alternative antimicrobial therapeutic [17]

This study represents a progression of our previous research as we aimed to overcome the problem of resistance by synthesizing new silver nanoparticles with novel structural and biological properties in the biomedical area. Our attention has been directed to examining the action of the AgNPs on factors of virulence in *Candida* species, as previously reported [2,3]. It is well known that the green synthesis silver nanoparticles exhibit several biological potentialities. For that in the present work, we reported new silver nanoparticles using *Aeonium haworthii* extract. 

The UV-Vis spectroscopy, FTIR, TEM and XRD analysis were undertaken to characterize the new biosynthesized silver nanoparticles from *Aeonium harworthii*. The biomedical and pharmaceutical behaviors were evaluated by testing their antioxidant, antimicrobial and antidiabetic capacities.

## 2. Materials and Methods

### 2.1. Plant Sample and Silver Nanoparticles Synthesis

The extremophile plant species *Aeonium harworthii* was collected from the Arid region located in Tunisia. The leaves of about (20 mg) were cleaned up by adding distilled water and allowed to dry at room temperature for several days, then the plant materials were ground to powder. Finally, a volume of 100mL of distilled water was added to the powder and agitated at 50 °C for 30 min. The resulting aqueous extract was filtered with Whatman paper and used for the green synthesis of silver nanoparticles.

The reaction mixture containing 5 mL of the aqueous extract and 5 mL of the AgNO_3_ solution at 1 mM was agitated for 4 min at room temperature. The biosynthesis of silver nanoparticles (AgNPs) was indicated by the color change detected from pale yellow to dark brown. The biosynthesized AgNPs were recuperated by centrifugation at 10,000 rpm for 15 min with Milli-Q water [3,4].

### 2.2. Characterization of Silver Nanoparticles

The structural properties of the biosynthesized AgNPs were conducted as previously described by [2,3] by different methods: UV-Vis spectroscopy using a 2802UV/VIS spectrometer (UNICO). In order to examine the shape and size of the AgNPs, we have used the transmission electron microscope (TEM), referred to as FEI Tecnai F20 S/TEM [27]. The X-ray diffraction (XRD) was performed on an X-ray diffractometer (D8 ADVANCE BRUKER) using Cu Kα radiation (λ = 1.5406 Å) [28]. The FTIR spectrum was determined in the range from 400 to 4000 cm^−1^ by using a Vrian FTIR640 spectrophotometer with KBr pellets [29].

### 2.3. Antioxidant Activity 

The DPPH (2,2-Diphenyl-1-picryl-hydrazyl) free radical scavenging activity of the AgNPs was examined by the DPPH reduction (1,1-diphenyl-2-picrylhydrazyl). Various AgNPs concentrations were prepared at mg/mL. The mix reaction contains 20 µL of each concentration and 200 µL of DPPH methanol solution. The negative sample using only the solvent was prepared in the same conditions. After incubation for 30 min, the absorbance was measured at 517 nm using UV-visible microplate spectrophotometry. The standard used was ascorbic acid. The scavenging activity was determined as a percentage of inhibition (PI) as recently described by Dridi and collaborators [2]. The IC50 was calculated using a calibration curve obtained from the antioxidant activity of each sample concentration.

### 2.4. In Vitro Antidiabetic Effect 

The in vitro antidiabetic action of the silver nanoparticles was evaluated by testing the inhibitory effect of alpha-amylase. The mixed reaction containing 500 µL of the sample (silver nanoparticles dilution), 500 µL Enzyme (alpha amylase 0.5 mg/mL, Sigma Aldrich Chemical Co, USA), was incubated at 25 °C for 10 min. Then, 500 µL of 1% starch solution was added and incubated for 10 min. After that 1 mL of DNS reagent was added to stop the reaction and heated for 5 min in boiling water, then cooled at room temperature and diluted by adding 5 mL of distilled water. The absorbance was recorded at 540 nm. For positive control, we replace the sample with 500 µL Acarbose. The percentage of inhibition was calculated as I% = Abs control − Abs sample/Abs control × 100 [30].

### 2.5. Microorganisms Strains Origins 

In order to investigate the antibacterial action of AgNPs, a list of clinical bacteria strains was used as *Klebsiella pneumoniae*, *Escherichia coli*, *Salmonella typhi*, *Staphylococcus aureus*, and *Micrococcus luteus.* The fungal strains used are Candida albicans and Candida nonalbicans (*Candida tropicalis*, *Candida glabrata*, *Candida sake* and two non-dermatophytic onychomycoses species *Aspergillus niger* and *Aspergillus terreus*.

### 2.6. Antimicrobial Detection 

The antibacterial and antifungal activities of the AgNPs were investigated by the agar well diffusion method as previously detailed [3]. Before use, each bacterial suspension was adjusted to 108 CFU/mL and the fungal suspension was adjusted to 10^5^ spores/mL. 

### 2.7. Minimum Inhibitory Concentration (MIC), Minimal Bactericidal Concentration (MBC) and Minimal Fungicidal Concentration (MFC) 

The broth dilution method was applied in 96 flat-bottom microliter plates and used to determine the MIC by visible examination of the microbial growth. The wells with no visible microbial growth (absence of turbidity) indicate the MIC value. To calculate MBC and MFC concentrations, we have used the methods detailed in [31,32]. The ratio MBC/MIC and MFC/MIC was calculated to examine the bactericidal or bacteriostatic action or fungicidal and fungistatic action of the AgNPs.

### 2.8. Effect of the Biosynthesized Silver Nanoparticles on the Factors Virulence of Candida Species

#### 2.8.1. Effect of AgNPs on Candida Growth and Morphogenesis

The reaction mix containing 1 mL of YM medium (Sigma Aldrich Chemical Co, USA), 20 µL of the silver nanoparticles at 0.1 mg/mL and 100 µL of each separate *Candida* species culture was adjusted to 10^5^ spores/mL. The incubation time was 48 h at 37 °C on a shaker. The inhibitory effect of the silver nanoparticles on Candida growth was calculated as Percent Candida survival = 100 × (A600 of Candida growth treated by AgNPs)/A600 of control tube (growth without the AgNPs) as reported by [2]. 

The effect of AgNPs on Candida cell morphology was examined by direct microscopic observation of 100 µL of cell culture at 400× and compared to untreated Yeast culture (control tube without the addition of AgNPs). Cotton blue was used as a colorant [33]. 

#### 2.8.2. Lipase and Proteinase Reduction

The effect of the silver nanoparticles on hydrolytic enzyme production (proteinase and phospholipase) was evaluated by using the method of Jin et al. [34], to determine the phospholipase activity (Pz) on the egg yolk agar medium. 

The bovine serum albumin medium (Sigma Aldrich, Taufkirchen, Germany), was used for the proteinase activity detection, by using the modified method of Staib as detailed by Mohandas and Ballal [35]. 

The surface of each specific medium was inoculated by 10 µL of *Candida* growth of 48 h at adjusted to 10^7^ CFU/mL. After that, the plates were incubated for 48 h at 37 °C. The diameter of colonies and the diameter of zone opacity were measured and the phospholipase activity (Pz) and the proteinase activity (Pz) were calculated from the Candida culture with or without the addition of the AgNPs based on the following formula:

Pz = (Candida colony diameter (in mm)/((Zone opacity + Candida colony diameter (in mm))(1)

### 2.9. Statistical Analysis 

The values present mean ± SEM. The generalized linear model (GLM) was used to compare groups of the SAS statistical program. Student Newman–Keuls SNK tests were used for multiple comparisons of means at 5%. Same letters are not significantly different, n = 3.

## 3. Results

### 3.1. Silver Nanoparticles Structural Characterization 

#### 3.1.1. Spectroscopic Analyses

**Figure 1:**
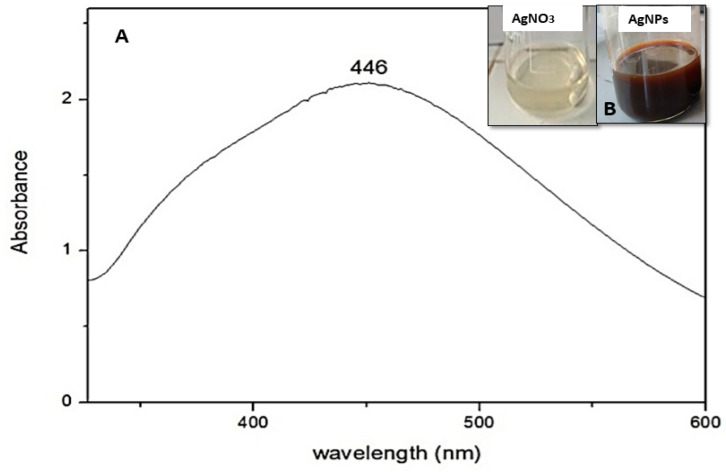
(**A**) Ultraviolet-visible absorption spectrum of silver nanoparticles and (**B**) Change of light brown color of AgNO_3_ solution to dark brown color of AgNPs.

UV-vis spectroscopy presents the first analysis used as an indicator for the production of stable silver nanoparticles. In the addition of the *Aeonium haworthii* leaf extract, the silver nitrate solution turned dark brown, indicating the formation of AgNPs (Figure 1B). 

The UV-Vis measurements present a fast and sensitive approach to select nanoparticle synthesis. The biosynthesized silver nanoparticles revealed a strong band absorbance at 446 nm (Figure 1A).

**Figure 2:**
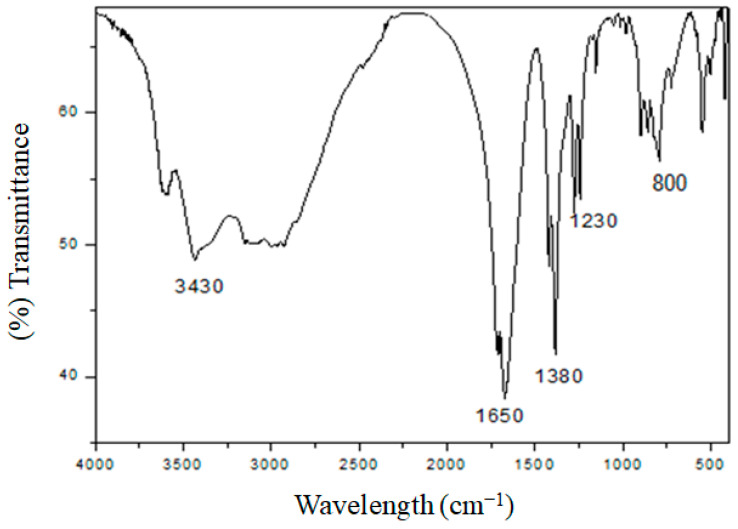
FT−IR spectrum of the AgNPs from *Aeonium haworthii* leaf extract.

Infrared spectroscopy (FT-IR) was performed to identify the bond linkages and functional groups associated with the *Aeonium haworthii* leaf extract treated with AgNO_3_. Identification of these groups is important to understand their involvement in the reduction process. The peak detection at 3430 cm^−1^ reflected the stretching vibration of OH groups specific to carboxylic acids. The peak absorbance at 1650 cm^−1^ was assigned to the carbonyl group [36,37]. Bands at 1380, 1230 and 800 cm^−1^ correspond to C–H, C–O and C–N functional groups, respectively [38]. The obtained results indicate the involvement of *Aeonium haworthii* leaf extract in the reduction in AgNO_3_ and prove to work as a capping agent for AgNPs. (Figure 2).

#### 3.1.2. Structural Study

**Figure 3:**
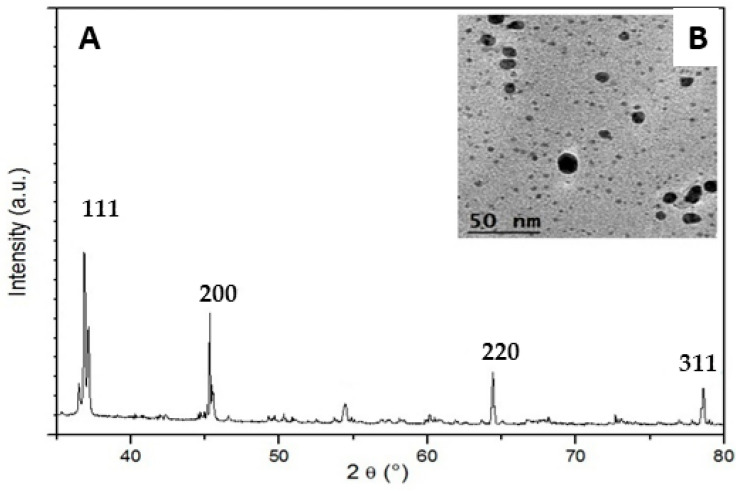
(**A**) XRD pattern and (**B**) TEM image of silver nanoparticles biosynthesized from *Aeonium haworthii* leaf extract.

The X-ray diffraction (XRD) pattern of the biosynthesized AgNPs shows four diffraction peaks (Figure 3A). These major peaks correspond to the (111), (200), (220) and (311) planes, respectively, and reflect the patterns of the face-centered cubic (fcc). The size of the silver nanoparticles will significantly influence the XRD peak patterns. The presence of various reducing agents in the leaf extract is responsible for the stabilization of AgNPs. Transmission Electron Microscopy (TEM) determined the size and shape of the biosynthesized AgNPs. The formation of silver nanoparticles as well as their morphological dimensions in the TEM study demonstrated that they have a spherical shape and their approximate size was found to be 35–55 nm with inter-particle distance (Figure 3B). 

### 3.2. Antioxidant Activity 

**Figure 4:**
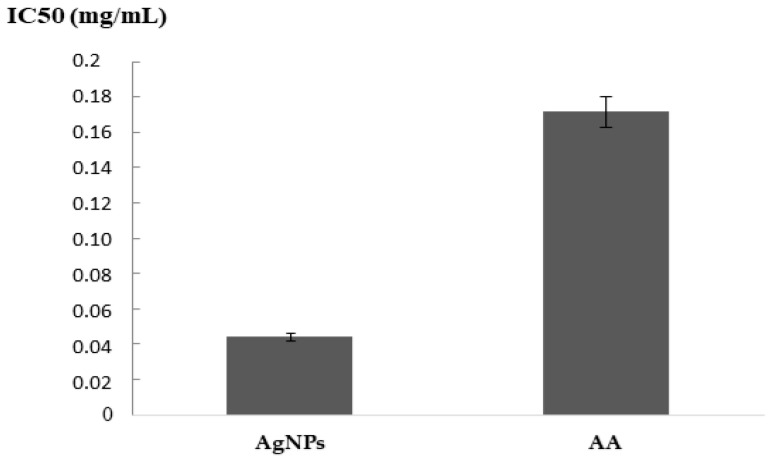
IC50 expressed in mg/mL of DPPH assay of the biosynthesized silver nanoparticles (AgNPs) and ascorbic acid (AA).

The IP was determined at various AgNPs concentrations. The IC50 has a value of 0.044 mg/mL, opposite to 0.1713 mg/mL given by ascorbic acid. The synthesized AgNPs possess an important antioxidant capacity to reduce the DPPH radicals, reflected by its superior antiradical activity (1/IC50) as compared to ascorbic acid (Figure 4).

### 3.3. In Vitro Antidiabetic Activity

**Figure 5:**
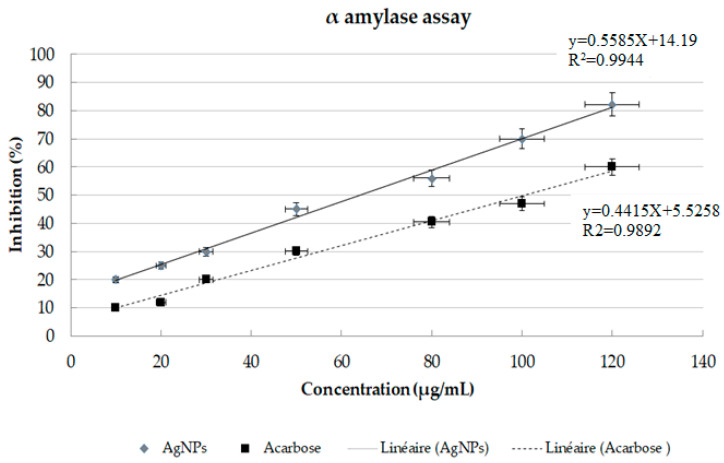
α amylase inhibitory effect of the AgNPs from *Aeronium harworthii* compared to the positive standard (Acarbose) used at 10 to 120 µg/mL. Values expressed as a percentage of inhibition. Error bars represent SE of the mean (n = 3) with a significant difference at *p* < 0.05.

The antidiabetic action of the silver nanoparticles was assessed by examining the α amylase inhibitory activity. Under in vitro conditions, in a dose depending manner, the biosynthesized silver nanoparticles from *Aeonium haworthii* displayed a superior inhibitory effect on the alpha-amylase as compared to the standard Acarbose. The highest enzyme inhibition was obtained at the concentration of 120 µg/mL for AgNPs and Acarbose, with 82.2% and 60%, respectively (Figure 5). The IC50 of AgNPs was about 62.84 µg/mL, as opposed to the IC50 of the control Acarbose with 100.73 µg/mL

### 3.4. Antimicrobial Screening by Agar Well Diffusion Method

The new biosynthesized silver nanoparticles displayed strong antibacterial and antifungal activities expressed by the observation of a maximum zone inhibition (ZI) expressed in mm. The great ZI of 18 mm was observed against the most sensitive strains of *Salmonella typhi*, followed by the strain *Klebsiella pneumoniae* with 17.5 mm. The ZI values do not exceed 15 mm against other bacterial strains. Mainly, the strongest antifungal activity detected by the AgNPs was observed against *Candida albicans* with a ZI of 18 mm, which was higher than the standard Fluconazole 25. For the Candida nonalbicans species high ZI values of 17.5 and 17 mm were noticed as compared to the standard Amphotericin B which gave less than 13.5 mm against Candida nonalbicans strains (Table 1).

**Figure 6:**
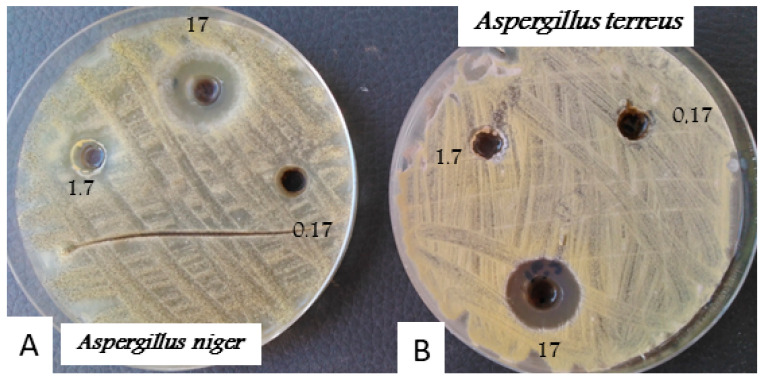
*Aspergillus niger* (**A**) and *Aspergillus terreus* (**B**) growth radial inhibition by the AgNPs. Values exhibited the AgNPs dilution expressed in µg/mL.

The AgNPs displayed a high antifungal effect on the radial growth of both tested non-dermatophytic onychomycoses species *Aspergillus niger* and *Aspergillus terreus.* The results show that the diameter of ZI ranges from 21 mm and 18 mm against *Aspergillus niger* and *Aspergillus terreus*, respectively (Figure 6).

### 3.5. Determinations of Minimal Inhibitory Concentration, Minimal Bactericidal Concentration and Minimal Fungicidal Concentration of AgNPs 

The Minimal Inhibitory concentration (MIC) of AgNPs ranged from 0.017 to 1.7 µg/mL. The Minimal Bactericidal Concentration (MBC) and Minimal Fungicidal Concentration (MFC) values vary from 0.017 to 3.4 µg/mL. The lowest MIC, MBC and MFC values proved the significant antibacterial and antifungal actions of the silver nanoparticles biosynthesized from *Aeonium haworthii.* The ratio MBC/MIC and MFC/MIC declared the bactericidal and fungicidal action of the AgNPs on bacterial and fungal strains (Table 2).

### 3.6. Silver Nanoparticles Effect’s on the Factors Virulence of Candida Species

#### 3.6.1. Effect of AgNPs on Candida Growth and Morphogenesis

The treatment of Candida growth with AgNPs amended the morphogenesis of treated Candida species. The addition of AgNPs stopped markedly the cell growth and limited the biofilm formation in all tested Candida species.

**Figure 7:**
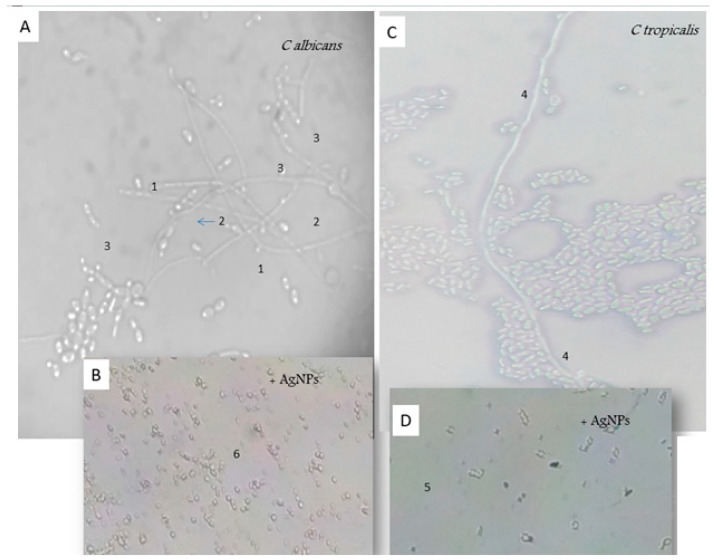
Comparative morphogenesis of *Candida albicans* growth without (**A**) and with (**B**) the addition of AgNPs and Candida tropicalis without (**C**) and with (**D**) the addition of AgNPs (×100 g). 1: germ tube, 2: clamydospore, 3: pseudofilaments, 4: real filaments, 5: blastospores and 6: altered blastospores.

The silver nanoparticles attack the morphogenesis of *Candida albicans* and *Candida tropicalis* strains as mentioned in Figure 7. The AgNPs block the way from blastospores to a filamentous form in both Candida species, since in the presence of AgNPs we observed only the blastospores (Figure 7B,D). Moreover, AgNPs showed a morphological alteration of the blastospores of *Candida albicans* (Figure 7B) as compared to the blastopores of *Candida tropicalis;* they reduce their germination. In the presence of AgNPs, *Candida albicans* was incapable to form a germ tube or chlamydospores, nor pseudofilaments; the absence of the filamentous morphogenesis makes *Candida albicans* not virulent to tissue invasion. The treated *C. tropicalis* with AgNPs (Figure 7D), lost its filamentous form as compared to the long filaments observed in the untreated *C. tropicalis* culture (Figure 7C).

#### 3.6.2. Effect of AgNPs on the Phospholipase and Proteinase Production by Candida Strains

**Figure 8:**
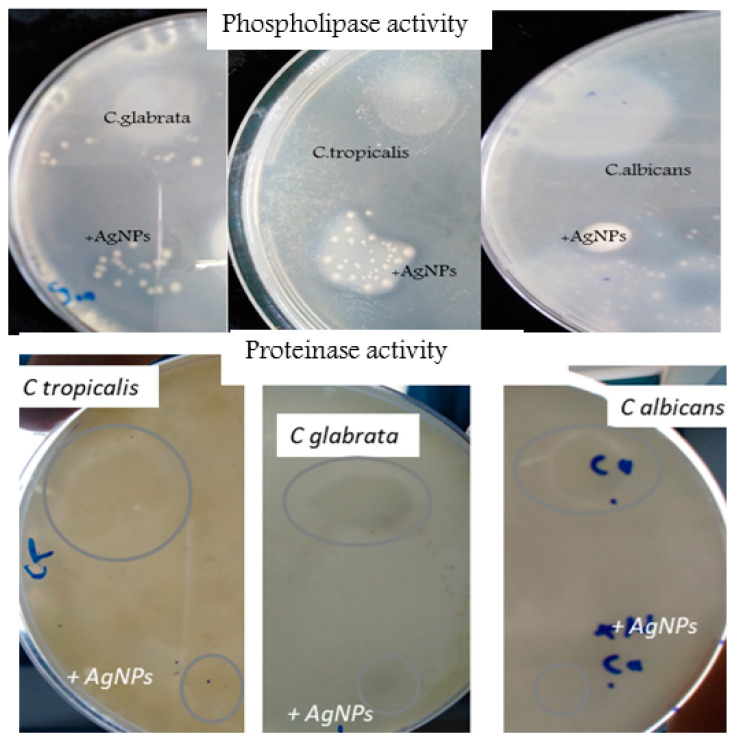
Comparative activities of phospholipase and proteinase produced by Candida species: *C. albicans*, *C. tropicalis*, *C. glabrata* in the absence and in the presence of AgNPs (+AgNPs). The observation of a clear zone around the Candida colony confirms the enzyme production.

Phospholipase and Proteinase productions of the Candida strains were evaluated in the absence of the silver nanoparticles (untreated) and in their presence (+AgNPs) (Table 3). The results have proven that the most common Candida species (*C. albicans*, *C. tropicalis* and *C. glabrata*), in candidose tissues were able to produce the enzymes key to virulence (phospholipase and proteinase) (Figure 8). The maximum enzyme production was observed by *C. albicans* in the absence of AgNPs and the Pz values ranged from 0.51 and 0.68 for the proteinase activity produced by *C. albicans* and *C. glabrata*, respectively.

For phospholipase activity the most productive Candida strain was *C. tropicalis*, in the absence of the silver nanoparticles. All Candida strains treated with AgNPs at 1.7 µg/mL showed a reduction in growth (Pz = 0.9) or a blockage of both enzyme productions (Pz = 1).

## 4. Discussion

Green technologies are gaining attention due to their effectiveness, non-toxicity and eco-friendliness. Silver nanoparticles are the most popular for their use in various areas. In view of the non-susceptibility of human pathogens to the currently commonly used antimicrobial drugs, it is necessary to design new natural antimicrobial molecules, among which silver nanoparticles are antibiotic agents [45].

Due to the relevant role of AgNPs in biological applications, some researchers directed the identification of toxicological examinations of NPs. Several in vivo models have been used in this way, such as [46], which demonstrates that the biodistribution, toxicokinetic and genotoxicity in murine models were related to the chemical structure of nanoparticles. Moreover, in in vivo models, the examination of the blood biochemistry and hematology, illustrates that the NPs show no systematic toxicity in rats and we have not detected any significant dose-related changes [47].

The new biosynthesized silver nanoparticles from *Aeonium haworthii* were characterized by a face-centered cubic (fcc) with an average size of 35–55 nm with inter-particle distance as determined by TEM. Various green synthesis silver nanoparticles were characterized by the same peak at 446 nm in the UV-visible spectrum, e.g., [48,49,50]. A similar average particle diameter as determined by TEM was found to be 35 nm for silver nanoparticles from *Malachra capitate* (L.) leaf extract [51]. The silver nanoparticles obtained from *Catharanthus roseus Linn*. G. Don leaf extract mentioned identical spherical shape nanoparticles with a diameter of 35–55 nm [52]. The inter-particles distance is an important factor in hotspot evolution and signal enhancement [53]. 

The phyto-nanoparticles seem to have high antioxidant properties. The most common and rapid method used for estimating the antioxidant activity was DPPH [54]. The high antioxidant activity of silver nanoparticles is explained by the presence of phenolic compounds, terpenoids and flavonoids. The current work is the first to choose the extremophile plant *Aeonium haworthii* for the biosynthesis of silver nanoparticles, since it may be richer in secondary metabolites that are required for extreme environment adaptation. These findings were established by various studies which highlighted the elevated antioxidant activity of AgNPs from plant species by DPPH [55,56]. In addition, in previous studies, we have indicated the strong antioxidant activity of the silver nanoparticles from the extremophile species *Scabiosa atropurpurea* subsp. *maritima* [3,57].

The inhibition of α amylase is one of the strategies for treating diabetes. Amylase inhibitors prevent the dietary absorption of starch, since they are blocking agents of starch, and then reduce the sugar levels in the blood. Moreover, it is well known that silver nanoparticles are represented as alpha-amylase inhibitors. The current study reported that IC50 obtained by Acarbose and AgNPs from *Aeonium haworthii* used at 120 µg/mL were 100.73 and 62.84 µg/mL. The work of Bagyalakshmi and Haritha [58] described that the IC50 obtained by AgNPs from *Pterocarpus marsupium* and Acarbose were 71.14%, and 21.88, respectively, at 700 and 180 µg/mL. The obtained results demonstrated the superior antidiabetic potential of our described AgNPs from *Aeonium haworthii*. 

The antioxidant and antidiabetic potentialities of silver nanoparticles may be due to the phytochemical composition of the vegetal extract required for the biosynthesis of silver nanoparticles by the green synthesis approach. Several works described the relationship between the antioxidant and the antidiabetic activities of silver nanoparticles and the plant extracts, e.g., [59].

Furthermore, several studies focused on the shape and size of AgNPs which varied according to the experimental methods used to influence their biological properties [60].

The antimicrobial effects of the NPs were famous in vitro. On the other hand, the in vivo animal models such as murine skin, liver and kidney damage profiles have proven the safety of the nanoparticles used as therapeutic agents. The in vivo toxicological study confirmed the safety of using the AgNPs as a promising antimicrobial in murine skin infection, by testing different parameters of renal function. The obtained results indicate no differences in the animals treated by AgNPs demonstrating that the NPs have no toxicity profiles, e.g., [46]. In addition, the work of Lee et al. [47], indicated that the nanoparticles improve oxidative stress, apoptosis and ion transport with slight toxicity by the examination of the organs of mice, hematology, biochemistry and histopathological measurements. In this context, we propose further in vivo toxicological studies to verify the safety of our new biosynthesized NPs before any biological applications. 

The mechanisms of action of AgNPs, were identified, such as the accumulation on the cell membrane, alteration of the bilayer integrity and the appearance of breaks. The fixation of AgNPs on the cell membrane avoids biofilm formation. The AgNPs penetrate into the cell and attack vital biomolecules leading to cell death. The current study highlighted the importance of new silver nanoparticles from *Aeonium haworthii* extract as antibacterial and antifungal agents due to the strong action against clinical strains proven by their low MIC, MBC and MFC values as compared to other published silver nanoparticles (see Table 2). 

In the present work, MIC values of the AgNPs Ah (from *Aeonium haworthii* extract) range from 0.017 to 1.7 µg/mL against bacterial and fungal strains. Other published AgNPs showed higher MIC values varying from 3.9 to 650 µg/mL related to AgNPs. The lower the MIC value the more efficient the antimicrobial drugs [39,40,41,42,43,44].

In the last decades, the invasion of candidiasis has increased dramatically, *Candida albicans* is the most common and *Candida glabrata* is the second infectious yeast species associated with different clinical types of candidiasis thus they gained the attention of mycologists and clinicians [61]. Moreover, due to the resistance of some *Candida albicans* and non*albicans* to current treatments, there is a need to discover a new active molecule. In this context, we have evaluated our new silver nanoparticles on *Candida* growth and virulence factors.

The results obtained in the co-cultural model (*Candida* + AgNPs), indicate the significant alteration of blastospores, and the absence of the filamentous morphogenesis which was required for biofilm formation. Several studies mentioned that silver nanoparticles highlighted the reduction in enzyme production as well as the elimination of biofilm formation in Candida strains [62,63]. Biofilm communities have been reported to be more virulent and resistant than free planktonic cells, and prevent attack by the host immune systems. Here, we describe new silver nanoparticles as anti-biofilm agents.

One of the most important factors of pathogenicity contributing to the virulence of *Candida* is enzyme production (proteinase, lipase and phospholipase). These enzymes play a crucial role in the adherence, penetration and invasion of the infected tissues. However, little is known about the effect of silver nanoparticles on the pathogenicity of *Candida albicans* and non*albicans*. For proteinase production, they facilitate adherence and the phenotypic switching of *Candida* by hydrolysis of the peptide bonds in proteins. Very little is known about enzyme inhibition by silver nanoparticles [64,65]. For this reason, in the present work, we highlighted the effect of silver nanoparticles on the growth and virulence of *Candida albicans* and nonalbicans factors belonging to *C. tropicalis* and *C. glabrata*, detecting the activities of phospholipase and proteinase. We described that a plate method allows for the rapid detection and measurement of the enzyme production by *Candida* species and the effect of the AgNPs on their production. Our results show that the ratio of colony diameter (Pz) correlates with the enzyme production by the *Candida* strains in the absence of silver nanoparticles. In addition, we highlighted that the maximum phospholipase and proteinase activities were reported by *Candida albicans* as compared to non*albicans* strains, these findings were also mentioned by the works of Ibrahim et al. [66] and Mane et al. [67].

Overall, the new silver nanoparticles exhibit superior antifungal action against *Candida* species growth and factors virulence as well as against the radial growth of non-dermatophytic onychomycoses *Aspergillus niger* and *Aspergillus terreus* at 0.17 and 1.7 µg/mL, respectively. These findings demonstrated the superior antifungal action of the described AgNPs as compared to other published works, for example, the MIC given by AgNPs against the radial growth of *Aspergillus flavus* is about 7.45 µg/mL [68]. In addition, Sodimalla and Yalavarthi, [69] described that silver nanoparticles from *Pseudomonas fluorescens* give a MIC of 150 µg/mL against *Aspergillus niger.*

## 5. Conclusions

This work presents an advancement in our research focused on the green synthesis of new silver nanoparticles from extremophile plant species. The green synthesis reported is a simple, fast, less energetic and eco-friendly approach as compared to others synthesis methods.

Here, we first reported the biosynthesized AgNPs from *Aeonium haworthii.* The new AgNPs possess a small size with 35–55 nm and a spherical shape. Regarding the robust anti-microbial, antioxidant and anti-diabetic potentialities obtained by AgNPs from *Aeonium haworthii* extract, we encourage their implication in the formulation of pharmaceutical and medical natural therapeutic drugs.

## Figures and Tables

**Table 1:** The antibacterial and anti-Candida activities of the biosynthesized AgNPs from *Aeonium haworthii*. Values present the zone inhibition against the tested strains expressed in mm. Means with the same letter are not significantly different from each other (*p* > 0.05).

Clinical Strains.	AgNPs	Standards	
Bacteria		Tobramycin	Vancomycin
*Escherchia coli* *Salmonella typhi* *Klebsiella pneumoniae* *Staphylococcus aureus* *Micrococcus luteus*	15 ± 0.5 ^c^ 18 ± 0 ^b^ 17.5 ± 0.5 ^b^ 14.5 ± 0.5 ^c^ 14 ± 0 ^cd^	12.5 ± 0 ^d^ 13 ± 0.5 ^d^ 19.5 ± 0.5 ^a^	12 ± 0 ^d^ 18 ± 0 ^b^
**Yeasts**		**Fluconazole 25**	**Amphotericin B**
*Candida albicans* *Candida tropicalis* *Candida glabrata* *Candida sake*	18 ± 0.57 ^c^ 17 ± 0.5 ^c^ 17.5 ± 0 ^c^ 17 ± 0 ^C^	13.5 ± 0 ^d^ 35 ± 1 ^a^ 20 ± 0 ^b^ 35 ± 0 ^a^	19 ± 0.5 ^b^ 10.3 ± 0 ^e^ 13.5 ± 0.5 ^d^ 10.5 ± 0.57 ^e^

**Table 2:** Comparative MIC, MBC, and MFC values of the biosynthesized AgNPs from *Aeonium haworthii* and other published AgNPs. Values are the average from triplicate experiments and expressed in µg/mL.

Pathogens *Bacterial Strains*	AgNPs [This Work]		AgNPs [39]	AgNPs [40]		AgNPs [41]	AgNPs [42]		AgNPs [43]	AgNPs [44]
	MIC	MBC	MIC	MIC	MIC	MBC	MIC	MBC	MIC	MIC
*Escherchia coli* *Salmonella typhi* *Klebsiella pneumoniae* *Staphylococcus aureus* *Micrococcus luteus*	0.017 0.017 1.7 1.7 0.17	0.017 0.017 1.7 3.4 0.34	12 . . 14 .	. . . . .	7.8 3.9 3.9 . .	7.8 7.8 3.9 . .	. . . 625 .	. . . 625	16 . . 32 .	. . . . .
***Fungal strains*** *Candida albicans* *Candida tropicalis* *Candida glabrata* *Candida sake* *Aspergillus terreus* *Aspergillus niger*	**MIC** 0.17 0.17 0.17 0.17 1.7 0.17	**MFC** 0.34 0.34 0.34 0.34 1.7 0.34	**MIC** . . . . . .	**MIC** 31.5 62.5 62.5 . . .	**MIC** . . . . . .	**MFC** . . . . . .	**MIC** . . . . . .	**MFC** . . . . . .	**MIC** . . . . . .	**MIC** 2.5 . . . . .

**Table 3:** Comparison of the virulence factor production by *Candida* species in the absence (Untreated) and in the presence of AgNPs (treated by the AgNPs) after 48 h of incubation at 37 °C.

Key Virulence		Hydrolytic Enzymes Production (Pz in mm)		Biofilm Morphogenesis
Candida Species		Phospholipase	Proteinase	
*C. albicans*	Untreated	Pz = 0.6	Pz = 0.51	(++++): Germ tube + chlamydospore s+pseudofilament
	AgNPs	Pz = 1 Negative	Pz = 1 Negative	(-) Altered Blastorpores
*C.tropicalis*	Untreated	Pz = 0.56	Pz = 0.62	(++++) Germ tube + real pseudofilament
	AgNPs	Pz = 0.9	Pz = 0.9	(-) blastospores
*C. glabrata*	Untreated	Pz = 0.74	Pz = 0.68	(+) blastospores
	AgNPs	Negative	Negative	(+) blastospores

Pz = 1 (negative); Pz: (0.9–0.99: +); Pz: (0.8–0.89: ++); Pz: (<0.7: ++++) Biofilm morphogenesis change (+++); absence of any morphogenesis change (-).

## Data Availability

Not applicable.
